# Sustained shoulder elevation posture: an under-recognized functional movement disorder phenotype

**DOI:** 10.1093/braincomms/fcaf454

**Published:** 2025-11-24

**Authors:** Alberto Albanese, Luigi M Romito, Paolo Amami, Daniela Calandrella, Tiziana De Santis

**Affiliations:** Department of Neurology, IRCCS C. Mondino Neurological Institute, Pavia 27100, Italy; Department of Neuroscience, Catholic University of the Sacred Heart, Milano 20123, Italy; Parkinson and Movement Disorders Unit, IRCCS Carlo Besta Neurological Institute, Milano 20133, Italy; Department of Neurology, IRCCS Humanitas Research Hospital, via Manzoni 56, Rozzano, Milano 20089, Italy; Department of Neurology, IRCCS Humanitas Research Hospital, via Manzoni 56, Rozzano, Milano 20089, Italy; Department of Neurology, IRCCS Humanitas Research Hospital, via Manzoni 56, Rozzano, Milano 20089, Italy

**Keywords:** functional movement disorders, dystonia, shoulder, abnormal posture

## Abstract

Patients with sustained shoulder elevation postures were observed over time in our movement disorders clinic and occasionally reported in literature as variants of dystonia or post-traumatic movement disorders. We retrospectively assessed the clinical records of patients in our movement disorders registry with sustained or fixed shoulder elevation. Their clinical phenomenology, response to treatment and precipitants were investigated. The patients underwent neurophysiologic, genetic and neuropsychologic tests. A PubMed search of cases with similar presentation was performed. Six patients fulfilled the inclusion criteria. Their phenomenology showed a sustained postural abnormality with elevation of one shoulder that often involves neighbouring regions; pain was a common accompanying feature; there were no alleviating manoeuvres, mirror or overflow phenomena. A recent preceding local trauma was reported by two patients; the onset was acute or gradual, progression was stable after initial worsening. There was poor benefit from oral medications; botulinum neurotoxin treatment improved pain and had little influence on postural abnormalities. Deep brain stimulation was ineffective in one patient; motor cortex stimulation caused partial or temporary improvement in two. All the patients received a diagnosis of functional movement disorder and met diagnostic criteria for functional neurological symptom (conversion) disorder with abnormal movements. The search strategies identified 19 publications reporting 75 similar cases, 75% of which were preceded by a minor traumatic injury. The motor abnormality responded poorly to oral medications, botulinum neurotoxin or physical therapy. We expand here on a peculiar phenomenology of sustained or fixed shoulder elevation that represents a recognizable syndrome with diagnostic and prognostic implications.

## Teaser

Albanese *et al*. report on a peculiar and rare phenotype observed in a tertiary movement disorders centre. The patients presented a functional movement disorder with sustained shoulder elevation posture, frequent pain, and no identifiable cause. Similar cases have been occasionally reported. Here the authors consistently systematize earlier and new observations.

## Graphical Abstract

**Graphical Abstract:**
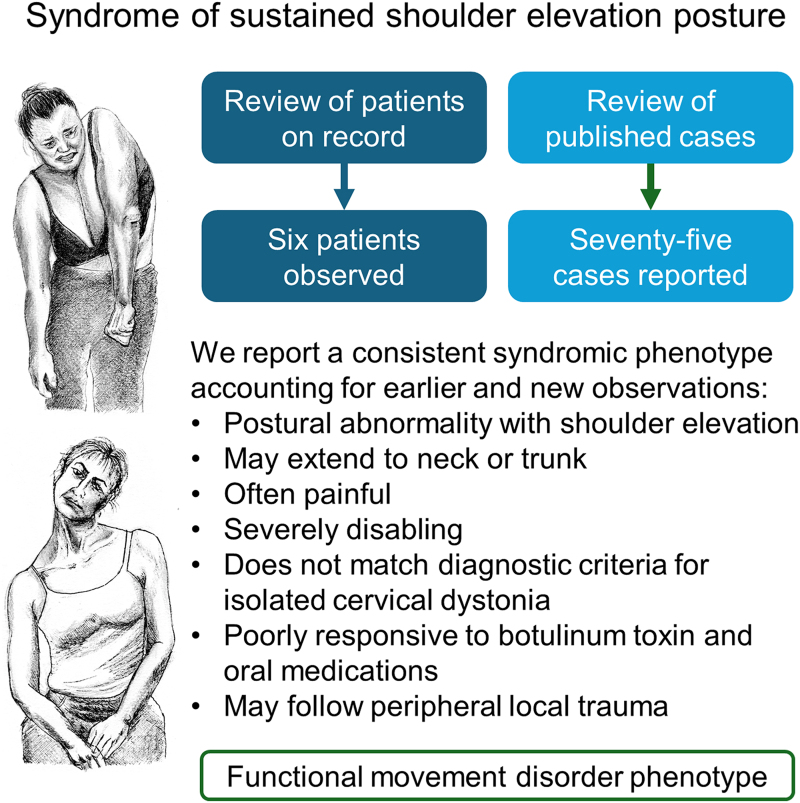

## Introduction

In a classic monograph, Denny–Brown retained that a fundamental feature in diseases of the basal ganglia was a disturbance of ‘attitude’ (term later replaced by ‘posture’), specifically ‘a tendency to patterned attitudes that at first were labile and gradually became more fixed’.^1^ He later called dystonia a postural abnormality causing ‘an abnormal degree of fixity of any attitude owing to sustained muscular contraction’.^2^ Marsden discouraged using the term dystonia to describe ‘immobile postures’,^3^ but did not propose an alternative terminology. The expression ‘fixed dystonia’ was later introduced to indicate ‘immobile dystonic postures that did not return to the neutral position at rest’.^4^ In most of these patients fixed postures occurred in the distal portion of a limb and were considered a functional movement disorder. This condition has also been observed in association with complex regional pain syndrome (CRPS).^5^

Fixed dystonia in a distal part of a limb, whether isolated or combined with CRPS, is a recognized entity affecting about half of the patients recorded in a functional dystonia registry.^6^ By contrast, cases of sustained or fixed proximal posturing are less frequent and have been only occasionally reported. Their course, prognosis and treatment options have not been systematized. We observed some patients with sustained postural elevation of the shoulder and were interested in the differential diagnosis and potential management options. This peculiar phenomenology raised our attention as we repeatedly saw these presentations over the years. We therefore reviewed the phenomenology of all the patients with a sustained shoulder elevation posture on record in our movement disorders registry and searched for similar cases in the literature. We describe here a consistent phenotype observed in some of our patients and also in other cases reported under different categories in earlier publications.

## Materials and methods

The clinical records of patients with dystonia referred to our Movement Disorders clinics between 2006 and 2023 were retrospectively analysed for inclusion. The available identified patients were contacted and summoned for reassessment; their clinical information, including neuroimaging, neurophysiology and genetic testing, was reviewed and updated. Most patients had video recordings that were repeated upon reassessment. The diagnosis was based on the full clinical evidence provided by direct examination and laboratory work-up until the last visit.

The patients were included if they had sustained or fixed shoulder elevation, with or without cervical or trunk involvement. Posturing was considered sustained when maintained at rest for prolonged periods of time but not fixed in response to passive movement, fixed when it could not be significantly changed by passive movement.^4^ The patients were excluded if they had orthopaedic or rheumatologic conditions affecting the shoulders. They all performed a detailed genetic panel for dystonia and other hyperkinetic movement disorders^7^; patients carrying gene variants likely pathogenic for a movement disorder were excluded. Patients with acquired dystonia (secondary to a known cause, such as brain lesion, neuroleptic assumption, Parkinsonism, etc.) were also excluded. All the patients provided informed consent to reproduction of their image for medical education; the study was approved by the Carlo Besta ethics committee.

Six patients (three men and three women) were included. They underwent a comprehensive physical and neurological examination; two had a complete psychiatric assessment. DSM-5 was used for diagnosing and classifying mental disorders.^8^ Neurophysiological assessment included electromyography, nerve conduction studies, and somatosensory evoked potentials. Brain MRI was performed in all patients; structural imaging of the spinal cord (either MRI or CT) was performed when clinically relevant. Cognitive assessments included a standard battery of tests evaluating memory, executive function, and visuospatial domains; psychological assessments included rating scales for anxiety, depression, body uneasiness, somatoform dissociation, alexithymia, and prior traumatic experiences. The Toronto Western Spasmodic Torticollis Rating Scale (TWSTRS)^9^ and the Psychogenic Movement Disorders Rating Scale^10^ were applied.

We conducted a literature review to analyse previous publications with a reported phenomenology comparable to that of the observed cases. A Medline search was conducted using the following search strategy: (‘Dystonic Disorders’ [MeSH Terms] OR ‘Dystonia’ [MeSH Terms] OR ‘Torticollis’ [MeSH Terms] OR ‘posture’ [Text Word]) AND ‘shoulder’ [Text Word] AND ‘humans’[Filter]. A second search additionally identified post-traumatic cases with similar phenomenology: (‘dystonia’[MeSH Terms] OR ‘torticollis’[MeSH Terms]) AND ‘traum*’[Title/Abstract]. There were no date limits, both searches were updated to February 2025 and restricted to languages known to the authors (English, French, German, Italian, and Spanish). Initial search results were screened by checking the title and abstract; to follow, full-text articles from the resultant list were evaluated for inclusion. Supplementary Fig. 1 reports the selection flow chart.

We subsequently reviewed the articles’ references and review papers to identify suitable reports not populated by the keyword-based search strings. Case descriptions were obtained from the published full-text, illustrations and video documentation where available.

We collected data related to: (i) the motor phenomenology, including body distribution, side of onset, temporal dimensions, and accompanying pain; (ii) the specifics of traumatic precipitants (either injury or surgery) that occurred before symptom onset; (iii) treatments received and response to therapy. We considered post-traumatic the cases where a physical or surgical injury occurred during the year preceding motor onset, according to proposed criteria.^11^

## Results

Six patients fulfilled criteria for inclusion in the study: their phenomenology is reported here and summarized in Table 1. Supplementary video clips provide visual examples of their phenomenology.

**Table 1:** Characteristics of patients reported in this series

General characteristics	Patient 1	Patient 2	Patient 3	Patient 4	Patient 5	Patient 6
Gender	F	F	F	M	M	M
Age at onset (years)	31	51	52	17	30	12
Symptom duration (years)	28	17	2	30	12	38
Hand dominance	Right	Right	Right	Right	Right	Right
DSM-5-TR^8^	Functional neurological symptom disorder (conversion disorder)					
Onset	Acute	Acute	Gradual	Gradual (arm), acute (shoulder)	Acute	Gradual
Site of motor onset	Shoulder	Shoulder	Shoulder	Arm, shoulder	Shoulder	Neck, shoulder, trunk
Side of onset	Left	Left	Left	Right	Left	Right
Pain	Yes	Yes	Yes	Yes	Yes	Yes
Preceding local injury or surgery	Yes^a^	Yes	No	No	Yes^a^	Yes
Diagnostic criteria						
Fahn and Williams^12,13^	Clinically established	Clinically established	Probable	Documented	Probable	Probable
Shill and Gerber^14^	Clinically possible	Clinically possible	Clinically possible	Clinically proven	Clinically possible	Clinically possible
Gupta and Lang^15^	Clinically established plus other features	Clinically established plus other features	Clinically established minus other features	Documented	Clinically established minus other features	Clinically established minus other features
Espay and Lang (dystonia)^16^	Definite 1, 2 Supportive 1	Definite 1, 2 Supportive 1	Definite 2 Supportive 1	Definite 2	Definite 1,2 Supportive 1	Definite 2 Supportive 1
Ratings						
TWSTRS scale^9^						
Torticollis severity score (0–35)	3	20	28	15	20	20
Disability score (0–30)	30	30	30	24	24	30
Pain score (0–20)	14	14	14	14	14	14
Psychogenic movement disorders rating scale^10^	23	22	18	18	25	23

. ^a^Trauma is consistent with criteria for post-traumatic movement disorder.^11^

### Case series

Patient 1 is a right-handed woman, with unremarkable personal or family medical history, who developed neck and left shoulder pain in 1990, at age 29. In 1994, she received a myotomy of the left anterior scalene muscle following an unconfirmed suspicion of thoracic outlet syndrome, without appreciable benefit. In 1995, she developed an intermittent painful elevation of the left shoulder that recurred multiple times during the day. Over the years, the elevation became gradually a painful fixed posture of shoulder elevation. Several botulinum toxin injection cycles did not relieve pain or improve the posture.

Over the next 8 years, abnormal posturing progressed distally, involving the left arm, which became extended and pronated; a clenched fist developed and abnormal postures also involved the neck and the trunk.

The patient was later seen by us. Her neck was tilted to the left, the left shoulder was elevated and rotated anteriorly, the left arm was abducted and intra-rotated, and her left fist was clenched. There were no dystonic fast movements, no overflow or alleviating manoeuvres. Several medications were ineffective, including abobotulinumtoxinA (up titrated to 1350 U), baclofen (up to 25 mg daily), trihexyphenidyl (up to 6 mg daily), or benzodiazepines. Under deep sedation with propofol the abnormal postures disappeared.

Aiming to improve what was considered a very disabling dystonia, in 2005, this patient received a bilateral globus pallidus internus (GPi) deep brain stimulation (DBS) implant that was also ineffective. In 2006, she received an epidural stimulation over the right primary motor cortex, over the central sulcus (four-plate Medtronic Resume electrode array). With GPi stimulation off, and cortical stimulation on (3.8 V, 60 Hz, 60 µsec, contacts −0 + 1–2), the patient had a gradual improvement over four months; pain and abnormal postures of the shoulder and trunk almost resolved. These treatments were documented in a case report.^17^ Her condition remained stable for 6 years after cortical implant. There were two documented relapses, when the cortical stimulator discharged in 2012 and 2017; in both instances the patient improved after replacement of the internal pulse generator. Over the following years, the abnormal posture of the left limb and trunk worsened, leading to reduced autonomy (not shown in the Supplementary Video). We later reconsidered the diagnosis based on interaction with colleagues and on reports of functional cases with similar phenomenology.^18^

**Figure 1:**
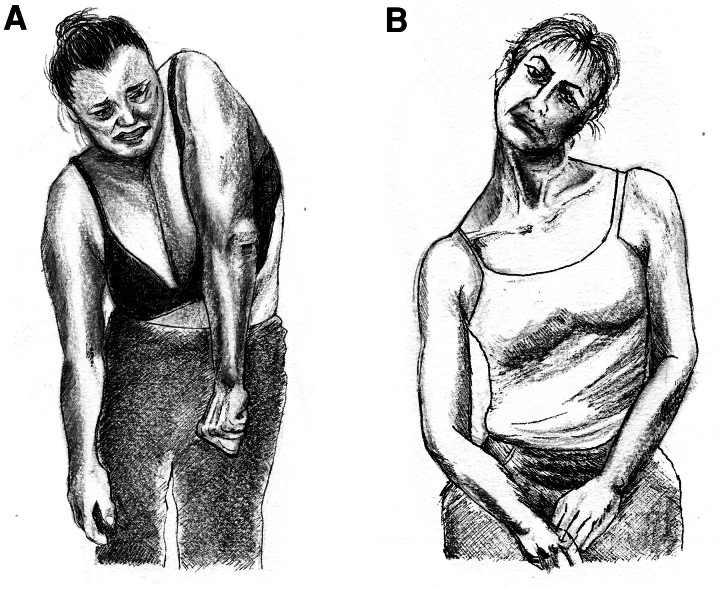
**Sustained shoulder elevation posture with prominent arm or cervical involvement.** These two patients depict two common presentations of sustained shoulder elevation posture. In Patient 1 (**A**), the left shoulder is elevated and the right shoulder is depressed; cervical involvement is negligible but the ipsilateral arm is fixed. In Patient 3 (**B**), the left shoulder is elevated and the right shoulder is depressed; there is cervical involvement, with ipsilateral head tilt. This cervical posture has been recognized as exemplary of functional cervical pseudo-dystonia^19^. Drawings by Dr. T. De Santis.

Her current condition is shown in Fig. 1A and Supplementary Video 1. With stimulation turned on (Segment 1), she has elevation and antero-rotation of the left shoulder associated with mild elevation, adduction, and intra-rotation of the left arm, as well as fixed finger flexion. Active movements were not possible, and passive range of motion was markedly reduced in the hand, absent in the arm and shoulder. With stimulation turned off (Segment 2), there is an increase of shoulder elevation and adduction, and a marked tilt of the trunk to the right. The patient reports pain and displays visible suffering.

Patient 2 is a right-handed woman with unremarkable family history. At age 18, she had a car accident with subsequent right shoulder trauma. At age 41, she received temporomandibular joint reconstruction due to an undocumented diagnosis of ankylosis.

At the age of 51, she suddenly awoke with a painful stiff neck and mild elevation of the left shoulder. This sustained posture later involved the left arm and the trunk. Treatment with abobotulinumtoxinA (up to 520 U) and diazepam (up to 4 mg daily) was not helpful. When she came to our attention in 2006, she had fixed painful mild elevation of the left shoulder and head tilt to the right, left sternocleidomastoid hypertrophy, no alleviating manoeuvres, mirror movements or overflow (Supplementary Video 2, Segment 1). Active movements were not possible.

Three years after onset, following the positive outcome of Patient 1, she also received an epidural implant over the left primary motor cortex (four-plate Medtronic Resume electrode array). Stimulation was delivered over the primary motor cortex parallel to the central sulcus (2.8 V, 60 Hz, 90 µsec, active contacts −2–3). This yielded a gradual recovery of symptoms. Twelve months later, she presented a brain infection near the epidural implant that was removed and later re-implanted. Subsequently, wide oscillating movements appeared when the right arm was outstretched: this tremor varied in amplitude, frequency and direction, was entrainable, and improved with distractibility manoeuvres. Fourteen years after the implant, the patient still reports that cortical stimulation attenuates her postures. Her current condition is shown in Supplementary Video 2 (Segment 2). There is a mild head tilt to the right that the patient can overcome with active movements, and a functional tremor of the right arm. Her left shoulder is mildly elevated. Stimulation was never discontinued, and three stimulator replacements were performed in 2014, 2018, and 2022.

Patient 3 is a right-handed woman who at age 52 noticed the gradual onset of a painful elevation of her left shoulder, followed by a progressive head tilt over a period of three months (Fig. 1B). Benzodiazepine treatment was unhelpful, and tizanidine (up to 8 mg daily) did not help. She came to our attention 2 years after onset, with a left shoulder elevation and head tilt to the left. There were no alleviating manoeuvres (Supplementary Video 3). Several abobotulinumtoxinA treatment cycles (up to 625 U) reduced shoulder and neck pain but were ineffective on the abnormal postures.

**Figure 2:**
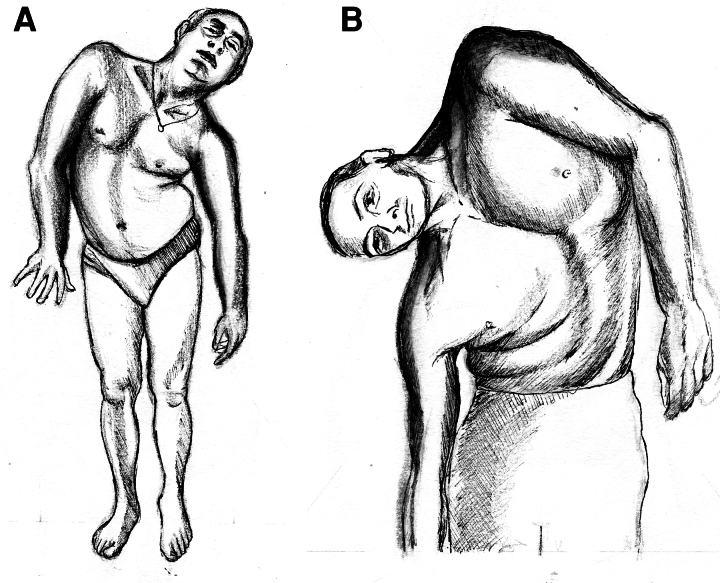
**Sustained shoulder elevation with prominent trunk involvement.** These two patients depict two less common presentations of sustained shoulder elevation posture. In Patient 4 (**A**), the right shoulder is elevated and the left shoulder is depressed; there is mild retrocollis and prominent trunk bending to the opposite side. In Patient 6 (**B**), the left shoulder is elevated and the right shoulder is depressed; there is mild head tilt to the opposite side and a remarkable trunk bending to the opposite side. Drawings by Dr. T. De Santis.

Patient 4 is a right-handed man, with unremarkable personal and family history and normal motor development. At age 17, he reported the onset of involuntary contractions in the right arm and received botulinum neurotoxin (BoNT) treatment for ∼20 years. At age 41, he gradually developed posturing of the right foot followed a year later by sudden elevation of the right shoulder. Psychiatric assessment reported a diagnosis of bipolar disorder; the patient was treated with valproic acid years after the posture appeared. Oral medications were unhelpful on the motor problem, including levodopa (up to 400 mg daily), trihexyphenidyl (up to 6 mg daily), clonazepam, and baclofen. BoNT injections (up to 180 incobotulinumtoxinA U) were also ineffective. The neurological examination showed abnormal postures with elevation of the right shoulder, mild right wrist abduction, inconstant trunk bending, and head tilt to the left. Active movements of the right arm had a normal range. There were mirror movements but no mirror dystonia or alleviating manoeuvres (Supplementary Video 4; Fig. 2A).

Patient 5 is a 42-year-old man with an unremarkable family history and normal motor development. At age 30, he reported an injury of the left splenius while practicing martial arts. Suddenly, he developed a perception of rigidity in the left shoulder that gradually resolved. When 41, he presented a sudden elevation of the left shoulder, associated with intense pain and head tilt to the left, with a direct ear-shoulder contact. One year later, this postural abnormality consisted of sustained left shoulder elevation, head tilt to the left, and left head rotation. Active range of motion was severely reduced in the neck, whereas there were no active or passive limitations in the right arm. His phenomenology varied over time, and he occasionally also had jerky head tremor, with variable amplitude and frequency (Supplementary Video 5). BoNT was not helpful.

Patient 6 is a 50-year-old man, with an unremarkable personal and family history and normal motor development. At age 12, he reported a head tilting to the right, following a punch to the abdomen struck by a schoolmate. The posture gradually worsened over the following 18 months, with elevation and abduction of the left shoulder, and a marked painful bending of the trunk to the right whenever he was standing. This postural anomaly improved partially in the sitting position (Supplementary Video 6; Fig. 2B). The patient had two periods of almost complete spontaneous remissions followed by relapses.

### Description of phenotype

The clinical picture of these patients was pleomorphic with some recognizable common features. They all had a painful postural abnormality with shoulder elevation and no identifiable cause. In some patients, shoulder elevation was the presenting feature, in others tremor or posturing in a limb preceded shoulder elevation. The patients were followed-up for an average of 21.2 years (8–34.4); their common features were: sustained postural abnormalities involving elevation of one shoulder, often involving the neighbouring regions (neck and trunk); frequent occurrence of local pain; absent or inefficacious alleviating manoeuvres; no mirroring or overflow; unremarkable family history, neuroimaging and genetic testing. This phenotype description was not observed in other patients in the registry, including those with genetic or acquired aetiology. Although in some patients the phenotype resembled that of cervical dystonia, it did not meet criteria for isolated cervical dystonia, particularly supportive criterion 1.^20^

The average TWSTRS pain score was 14/20; disability score was 28/30. Symptom onset varied from acute in three patients (1, 2, and 5), to gradual in the other three (3, 4, and 6). Two patients reported a preceding local trauma, physical injury in one, surgery in the other. In two patients, a local trauma had occurred 33 and 43 years before the onset of the abnormal shoulder posture. Other postural abnormalities were inconsistent: in three patients, the head was tilted to the opposite side of shoulder elevation; in two, the head was straight compared to the shoulders; and in one the head was tilted towards the side of shoulder elevation. These sustained or fixed postures remained individually consistent over time. The trunk was at times bent towards the opposite side of shoulder elevation.

Structural imaging and neurophysiological studies were normal in all patients. There were no reported cognitive difficulties. Psychiatric history was remarkable in one patient. Rating scales revealed no dissociative symptoms, difficulties in emotional regulation, or alexithymia, while body uneasiness and anxiety were common findings. All patients met DSM-5 criteria for functional neurological symptom (conversion) disorder with abnormal movement.^8^

BoNT treatment was administered to all and inefficacious on motor phenomena, but relieved pain in three patients. Bilateral GPi DBS was ineffective on Patient 1; motor cortex stimulation was partially effective on Patients 1 and 2. Table 1 summarises the main clinical characteristics. All the patients received a diagnosis of functional movement disorder supported by three sets of diagnostic criteria; they also matched some phenotype-specific diagnostic criteria for dystonia (Table 1). Their average FMDRSS score was 21.5.

### Earlier reports

Our search strategies identified a total of 3453 publications (Supplementary Fig. 1). After selection, we included 19 publications encompassing a total of 75 cases whose clinical description reported a sustained or fixed shoulder elevation (Table 2; Supplementary Table 1). One of the included publications was an earlier report of Patient 1.^17^ Seven patients were described as single case reports; 67 were part of small case series, often mentioning a preceding trauma. One patient was included as part of a series describing fixed dystonia (Patient 4 in the prospective cohort^4^). The search strategy also identified ten earlier case descriptions with a suggestive phenomenology that was however insufficiently demonstrative of the condition (Supplementary Table 2).

**Table 2:** Earlier publications reporting patients with sustained shoulder elevation

Earlier description	Sustained shoulder elevation	Presumptive aetiology^a^	Pain^a^	Precipitating factors (delay)^a^	Progression and outcome^a^	Follow-up^a^	References
Cervical dystonia with abrupt onset after neck trauma	3 of 6	Organic non-dystonic torticollis	Neck, shoulder	Peripheral or central trauma (1–4 days)	Some improvement with BoNT	Up to 38 years	Truong et al.^21^
Cervical dystonia with acute onset after mild neck trauma	1 of 5	Organic dystonia precipitated by peripheral trauma	NR	Peripheral trauma (24 h)	Some improvement with BoNT and physical therapy	<3 years	Goldman and Ahlskog^22^
Cervical dystonia with onset after mild trauma	8 of 15	Non-dystonic spasm or torticollis; presumptive psychogenicity in <4-week cohort	Neck ± shoulder	Peripheral or central trauma (<4 weeks or >3 months)	Persistent; some improvement with BoNT	1–13 years	Tarsy^23^
Shoulder dystonia with onset shortly after minor shoulder injury	2 of 2	Organic, post-traumatic dystonia	Shoulder	Peripheral trauma (few days)	Persistent	2 years	Thyagarajan et al.^24^
Focal shoulder (non-cervical) dystonia after minor shoulder trauma	2 of 2	Organic dystonia after musculoskeletal injury	Shoulder	Peripheral trauma (<1 day to 9 months)	Persistent, some improvement with BoNT	1–8 years	Hollinger and Burgunder^25^
Focal shoulder elevation (non-cervical) dystonia	13 of 13	Organic dystonia after peripheral injury	Shoulder	Peripheral trauma, heavy labour, radiculopathy	Persistent, some improvement with BoNT	0.5–7 years	Wright and Ahlskog^26^
Shoulder girdle dystonia and nuchal pain following cervical disc surgery	13 of 13	Organic dystonia after peripheral injury	Neck, arm	Cervical disc surgery	NR	NR	Becker et al.^27^
Painful torticollis shortly after minor local injury	15 of 16	Psychogenic	Neck, shoulder	Minor local injury (few hours)	Persistent, inconstant mild improvement with BoNT	2–12 years	Sa et al.^28^
Cervical dystonia after cervical injury	2 of 9	Peripheral trauma	Neck	Local injury (few hours)	Persistent, mild improvement with BoNT	1–5 years	Frei et al.^29^
Cervical dystonia after mild local trauma	6 of 16	Post-traumatic dystonia	NR	Peripheral trauma (<4 weeks)	Persistent, pain improvement with BoNT	3–15 years	O'Riordan and Hutchinson^30^
Fixed dystonia in any body region	1 of 103	Psychogenic dystonia	Neck	Undetailed surgery	Persistent	4 years	Schrag et al.^4^
Post-traumatic dystonia unresponsive to DBS	1 of 1	Organic painful tonic dystonia	Foot	Car accident (years)	Persistent, unresponsive to GPi DBS	19 months	Capelle et al.^31^
Shoulder dystonia after minor shoulder trauma	1 of 1	Acquired focal dystonia	Shoulder	Fall (one year)	Persistent, mild improvement with BoNT	2 months	Carroll et al.^32^
Cervical dystonia after cervical spine surgery	1 of 1	Post-traumatic dystonia	Neck	C2-C6 corpectomy (6 weeks)	Resolution with BoNT	6 months	Takemoto et al.^33^
Fixed shoulder dystonia^b^	1 of 1	Primary dystonia	Shoulder	NR	Persistent, unresponsive to GPi DBS	22 months	Romito et al.^17^
Fixed shoulder dystonia after mild shoulder trauma	1 of 1	NR	Shoulder	Peripheral trauma (8 months)	Resolved after 4 months	4 months	Drouet et al.^34^
Peripherally induced movement disorder	2 of 2	Peripherally induced dystonia	NR	Peripheral or central injury (<1 year)	Resolution (<1 day), persistent (one patient)	>9 months	Jankovic^35^
Fixed shoulder and trunk dystonia after violent shoulder trauma	1 of 1	NR	Shoulder	Peripheral trauma (<1 day)	Persistent, improved with intravenous steroid treatment	14 months	Hassan et al.^36^
Shoulder dystonia following trauma in the arm	1 of 1	NR	Neck	Peripheral trauma (3 years)	Persistent, improved with BoNT treatment	2 years	Vasileiadis et al.^37^

BoNT, botulinum neurotoxin; GPi, globus pallidus internus; NR, not reported. ^a^Refers to included patients only. ^b^Refers to case 1 in current series.

In earlier publications this phenotype was often described as a peculiar form of dystonia, with varied denominations: post-traumatic torticollis,^21^ post-traumatic cervical dystonia,^22,23,29,38^ cervical dystonia induced by cervical spine surgery,^33^ post-traumatic shoulder dystonia,^24,25,32^ shoulder elevation dystonia,^26^ shoulder girdle dystonia,^27^ post-traumatic painful torticollis,^28^ fixed dystonia.^4,17^ Non-dystonic denominations were also used: non-dystonic torticollis,^21^ peripherally induced movement disorder,^35^ abnormal shoulder elevation,^39^ Brancusi’s suffering.^40^ The expression Malevich’s shoulder has also been used to describe a similar phenomenology observed in Parkinson’s disease.^41^ These varied denominations are interesting, as each underlines a different phenomenological aspect of the condition. Aetiology was considered functional in two case series,^4,28^ organic or post-traumatic in the others (Table 2).

Precipitating factors were reported by several studies (Table 2). In 55 patients (73%), these consisted of a usually minor single traumatic injury, most frequently involving the neck or the shoulder. A preceding cervical disc surgery was reported in 15 patients.^4,27,33^ Distant injuries were also mentioned: in one case trauma involved the foot, in one the jaw and in two the head. A relapse after a second injury was reported in one case.^26^ Although traumatic events were considered precipitating factors, their reporting was generally incomplete and missed relevant details, such as the dynamic of trauma and whether it was direct or indirect. In most cases, the precipitating trauma consisted of a motor vehicle accident or an accident at work. The follow-up was generally not accurately reported.

Age at onset varied from adolescence (five patients, 6%), to early adulthood (39 patients, 44%), middle adulthood (14 patients, 19%), and late adulthood (two patients, 3%). In 15 cases, age at onset was unreported. The onset was acute in 48 patients, subacute (over days) in two, gradual (over months) in nine, and unreported in 16. Fifty-four patients had visible shoulder muscle hypertrophy; alleviating manoeuvres were occasionally mentioned.

BoNT treatment was either reported as poorly effective or as ineffective, with some reports mentioning a relief of pain and posture, and others reporting only pain alleviation. In few patients, however, pain was mentioned to worsen after BoNT treatment.^23,28^ Oral therapy with benzodiazepines and anti-cholinergics was ineffective in all treated patients. Amytal was occasionally administered to reduce the abnormal posture, with some reported efficacy lasting after the infusion.^28^ Physical therapy was either ineffective or partially effective. Steroid therapy was administered to two patients and considered efficacious in one.^29,36^ GPi DBS was ineffective,^17^ as well as GPi combined with thalamic stimulation.^31^ Improvement after cortical stimulation was reported in one case.^17^

Some earlier observations suggested a functional or psychogenic aetiology, and assessed the patients’ psychological status.^42^ Several earlier publications emphasized the possible role of psychological factors in aetiology or in the persistence of elevated shoulder posture, pain and associated disability.^4,22,26,28,29^ A psychological assessment was available for 13 patients.^4,22,28^

## Discussion

We report here a rare sporadic condition observed in six patients from a series of more than 1200 dystonia cases. They developed a sustained (occasionally fixed) posture with shoulder elevation and variable involvement of the neck or trunk that did not benefit from oral medications for dystonia. The core presentation was consistent across all, although its severity varied from mild shoulder elevation with head tilt to more severe postures involving the trunk. The condition generally caused severe disability, often out of proportion to the observed motor disturbance. Few of these cases were initially considered functional cervical dystonia variants. In our patients the TWSTRS scale showed high scores for disability and pain, whereas cervical motor severity scores varied (Table 1). Tremulous oscillations of the head, trunk, or limbs were observed in some patients. Functional impairment was significant in all, and influenced the patients’ working ability and social life, motivating them to experiment interventional approaches. BoNT injections usually relieved the associated pain but were poorly effective on postural abnormalities, in keeping with earlier reports that BoNT is not better than placebo in relieving functional movement disorders.^43,44^ This aspect highlights a difference from cervical dystonia, whose motor features typically improve with BoNT treatment.

The condition reported here is different from common orthopaedic or rheumatologic shoulder problems that are often associated with local pain.^45^ Differential diagnosis includes adhesive capsulitis, scoliosis, muscle imbalances, shoulder dislocations, accessory nerve palsy, and congenital conditions, such as Sprengel deformity. Other movement disorders featuring fixed postures should also be considered (e.g. *ATP1A3*-related dystonia). This differential is particularly relevant when the patients are seen outside a movement disorders setting. Repeated shoulder jerks observed in patients with functional dystonia, which have a semi-rhythmic appearance, should also be considered in the differential diagnosis.^12,46^ Shoulder dislocation has been reported in some of these cases, but not in patients with sustained shoulder elevation posture.

A recent consensus indicates that the phenotype reported here does not tally with the definition of cervical dystonia, a more common condition primarily associated with abnormal head and neck postures that can extend to the shoulders.^20^ In the past, milder presentations of sustained shoulder elevation posture have been considered functional variants of cervical dystonia with a characteristic phenotypic presentation of predominant laterocollis, ipsilateral shoulder elevation, and contralateral shoulder depression.^47^ This sketchy description is consistent with the phenotype observed in Patients 3 and 5, but not with that of Patients 1, 2, 4, and 6 of this series. We observe here that the head tilt can be on either side relative to the elevated shoulder, or may not be appreciable, emphasizing that the syndrome of sustained shoulder elevation posture is pleomorphic. Contralateral shoulder depression is not mandatory and there are cases where cervical involvement is limited or absent. Some earlier observations evidenced that these patients did not present features of cervical dystonia^25^ and highlighted the difference with shoulder elevation associated with cervical dystonia.^26^ To facilitate a proper recognition of these patients, it seems appropriate to identify sustained shoulder elevation posture as the key phenomenology. A further distinction is that BoNT, a first-line treatment option for cervical dystonia, does not improve the postural abnormality of these patients. It is also of note that the observed postural abnormalities are in most cases inconsistent with the general definition of dystonia and often lacked several typical features, such as dystonic movements, alleviating manoeuvres, or voluntary activation.^48^

Patients with sustained shoulder elevation posture have been regularly reported since the late 1980s; our search identified 75 previously published similar cases and ten suggestive reports. The number of publications peaked in 1998 and 2003. Familial occurrence was never mentioned. These descriptions also revealed a variable degree of severity and body distribution with sustained shoulder elevation posture as a constant feature^17,24-27,32,34,37,39^ Other reports noted a concomitant cervical involvement and considered this phenotype a variant of cervical dystonia.^21-23,28,29,38,47^ There is agreement that BoNT can only be partially effective.^21,23-26^ We did not observe involvement of the facial district in our patients, but found one published case with elevated shoulder posture and functional facial movements.^49^ It has been argued that earlier descriptions of functional movements of the face may have overlooked concurrent sustained shoulder elevation postures.^49^

This syndrome has been compared to postural abnormalities seen in patients with causalgia-dystonia or CRPS and dystonia. Fixed posturing (often called ‘dystonic’) is a recognized phenomenon in CRPS,^3,50,51^ with typical involvement of a distal extremity and occasional spread towards the proximal shoulder or pelvic girdle.^5^ It has been observed that features of CRPS may develop in patients with sustained shoulder elevation posture, either on the same side^29,36^ or in a different limb.^31^ However, patients with this condition were rarely reported to develop signs of microvascular dysregulation or focal dysautonomia that can be observed in CRPS.^52^ Whether sustained shoulder elevation posture may be an initial or incomplete variant of CRPS is open to discussion. The temporal relationship between the onset of pain, sympathetic abnormalities, and abnormal posturing has not been studied in detail in these patients. It is however recognized that fixed postures and pain can develop after prolonged immobilization, such as casting,^53^ as well as in patients with CRPS.^5^ Peripheral injuries are a recognized precipitant of CRPS,^54,55^ as well as of sustained shoulder elevation posture.^21-27,29-32,35^ The specifics of the reported traumatic injuries are not well characterized; their temporal relationship with symptom onset is variable, as are their reported severity and causes, including a variety of events, such as car accidents, work-related injuries, falls, surgical interventions, etc. A precipitating role of peripheral trauma has been postulated for different movement disorders, including those with a dystonia phenotype^55-58^ and genetic pre-disposition has been considered a predisposing factor. It has been reported that physical injury can indeed occur before symptom onset in patients with motor or sensory conversion symptoms^59^ as well as in those with functional movement disorders.^60^

Peripheral trauma, whether physical injury or surgery, is a recognized precipitant of functional movement disorders, and has been reported in patients with a diagnosis of functional dystonia.^57,61^ In the case of post-traumatic movement disorders, it has been considered that a peripheral injury must be severe enough to cause local symptoms for at least two weeks, and that the onset of the movement disorder should be within a reasonable time frame (up to 1 year) after the injury and anatomically related to the site of the injury.^11^ A preceding traumatic event consistent with criteria for post-traumatic movement disorder was reported by 33% of our patients, who were selected based on phenomenological criteria, and by 80% of the patients reviewed, who were predominantly reported as post-traumatic events.

Fixed postures with acute or subacute onset in adulthood are a consistent presentation of functional movement disorders.^6^ Most of the patients reviewed here had an acute onset, while in others the onset was gradual. In some patients, postures were sustained rather than fixed. The course was usually progressive during the first months, spreading to contiguous body regions; it then became static in almost all patients, with few variations. All our patients received a diagnosis of functional movement disorder and fulfilled current diagnostic criteria, but did not meet all the phenotype-specific criteria required for the diagnosis of clinically definite functional dystonia,^16^ suggesting that the term dystonia may not be appropriate for these cases.

The phenomenological spectrum of functional movement disorders is rapidly expanding. Some of the patients reported here presented positive diagnostic signs described for functional movement disorders and particularly pseudo-dystonia, including paradigmatic cervical posturing, resistance to passive movements, or distractibility.^19,62^ Sustained or fixed shoulder elevation is a diagnostic red flag raising a suspicion of functionality that requires focused assessment. Cases with relevant trunk involvement (33% in our series) may pose diagnostic uncertainty and require repeated observations and additional work-up. Many of these patients had increased anxiety and body image disturbance. However, only two of our patients received a formal psychiatric evaluation, which is not standard practice in the setting of neurology. We also acknowledge that the retrospective design of this analysis has some inevitable limitations, particularly recall and selection biases. This may have potentially underestimated psychiatric comorbidities as well as the incidence of the reported phenotype. We highlight the importance of a multi-disciplinary team in the management of patients with functional movement disorders. Body image disturbances have been reported in patients with fixed dystonia and type 1 CRPS.^63^ The role of psychiatric precipitants and comorbidities in functional movement disorders is still poorly understood. Psychological ‘conflicts’ are not mandatory for the diagnosis and are not mentioned in DSM 5 criteria of conversion disorders.^8^ A somatoform profile has been recognized in some patients with painful shoulder elevation posture.^28^ Other studies suggested a normal psychological profile in patients with functional movement disorders,^64^ but a high prevalence of psychological stressors and dissociative correlates in functional dystonia.^65^ It has also been reported that affective disorders are more common in fixed dystonia compared to non-functional (formerly organic) mobile dystonia.^4^ ^28^ Stressful events and maltreatment can facilitate the onset of functional movement disorders.^66^

Recognition of sustained shoulder elevation posture depends on careful history taking and examination, because there are no biological markers. Positive signs include a sustained or fixed posture with shoulder elevation and associated pain. A traumatic precipitant may be appreciated. The condition is disabling, limiting usage of at least one upper limb and influencing the overall patient functioning. We believe that increased recognition of this specific functional phenotype may help ascertain its prevalence and contribute to improving its management.

## Supplementary Material

fcaf454_Supplementary_Data

## Data Availability

Clinical data may be provided upon request in anonymous format.
