# Supplementary material for: Sustained shoulder elevation posture: an under-recognized functional movement disorder phenotype
Source: Brain Commun. 2025 Nov 24;7(6):fcaf454. doi: 10.1093/braincomms/fcaf454 (PMC12836432; doi:10.1093/braincomms/fcaf454)
Supplement: fcaf454_Supplementary_Data [file fcaf454_Supplementary_Data.zip › Supplementary material.pdf]

Sustained shoulder elevation posture: an under-recognized functional movement disorder phenotype

Alberto Albanese MD,[1,2] Luigi M. Romito, MD, [3] Paolo Amami, PhD, [4] Daniela Calandrella, MD, [4] Tiziana De Santis, MD [4]

[1] Department of Neurology, IRCCS C. Mondino Neurological Institute, 27100 Pavia, Italy

[2] Department of Neuroscience, Catholic University of the Sacred Heart, 20123 Milano, Italy

[3] Parkinson and Movement Disorders Unit, IRCCS Carlo Besta Neurological Institute, 20133 Milano, Italy

[4] Department of Neurology, IRCCS Humanitas Research Hospital, 20089 Rozzano, Milano, Italy

Supplementary video legends

Supplementary video 1. Patient 1 has fixed elevation of the left shoulder, adduction and intra-rotation of the upper left limb, and clenching of the left fist. There is no significant cervical involvement. Cortical stimulation of the right primary motor cortex is turned on in segment 1. With stimulation turned off the picture becomes more prominent and a rightward bending of the trunk is evident (segment 2).

Supplementary video 2. Patient 2 has fixed elevation of the left shoulder, head tilting to the right, and depression of the right shoulder (as observed in preoperative condition; segment 1). Fourteen years after continuous cortical stimulation on the right primary motor cortex, the shoulder elevation is reduced, and a functional oscillation of the right arm has appeared (segment 2).

Supplementary video 3. Patient 3 has a fixed elevation of the left shoulder and head tilt to the left. As a result, the right shoulder is depressed. This phenomenology has been described as a paradigmatic phenotype of functional cervical pseudo-dystonia.

Supplementary video 4. Patient 4 has a fixed elevation of the right shoulder, ulnar deviation of the right wrist, and trunk bending to the left. At times, the head is extended. The abnormal trunk posture gets worse as he stands.

Supplementary video 5. Patient 5 has sustained left shoulder elevation; head is tilted to the left and mildly rotated. There are oscillations of the head and trunk with variable amplitude and frequency that were attenuated when the patient was distracted.

Supplementary video 6. Patient 6 presents with fixed elevation of the left shoulder associated to a remarkable trunk bending to the right whenever he stands. The head is mildly tilted to the right; his posture changes only when he sits or stands. Trunk involvement is more pronounced here than in patient 4.

Supplementary figures

Supplementary figure 1. PRISMA flow diagram of the different phases of the search strategy. It maps out the number of identified records, included and excluded, and the reasons for exclusion. 19 publications were eventually included in Supplementary Table 1 (documented phenotype) and 10 were included in Supplementary Table 2 (suggestive phenotype). One publication contained both a documented and a suggestive phenotype; hence, in total 27 articles were eligible.

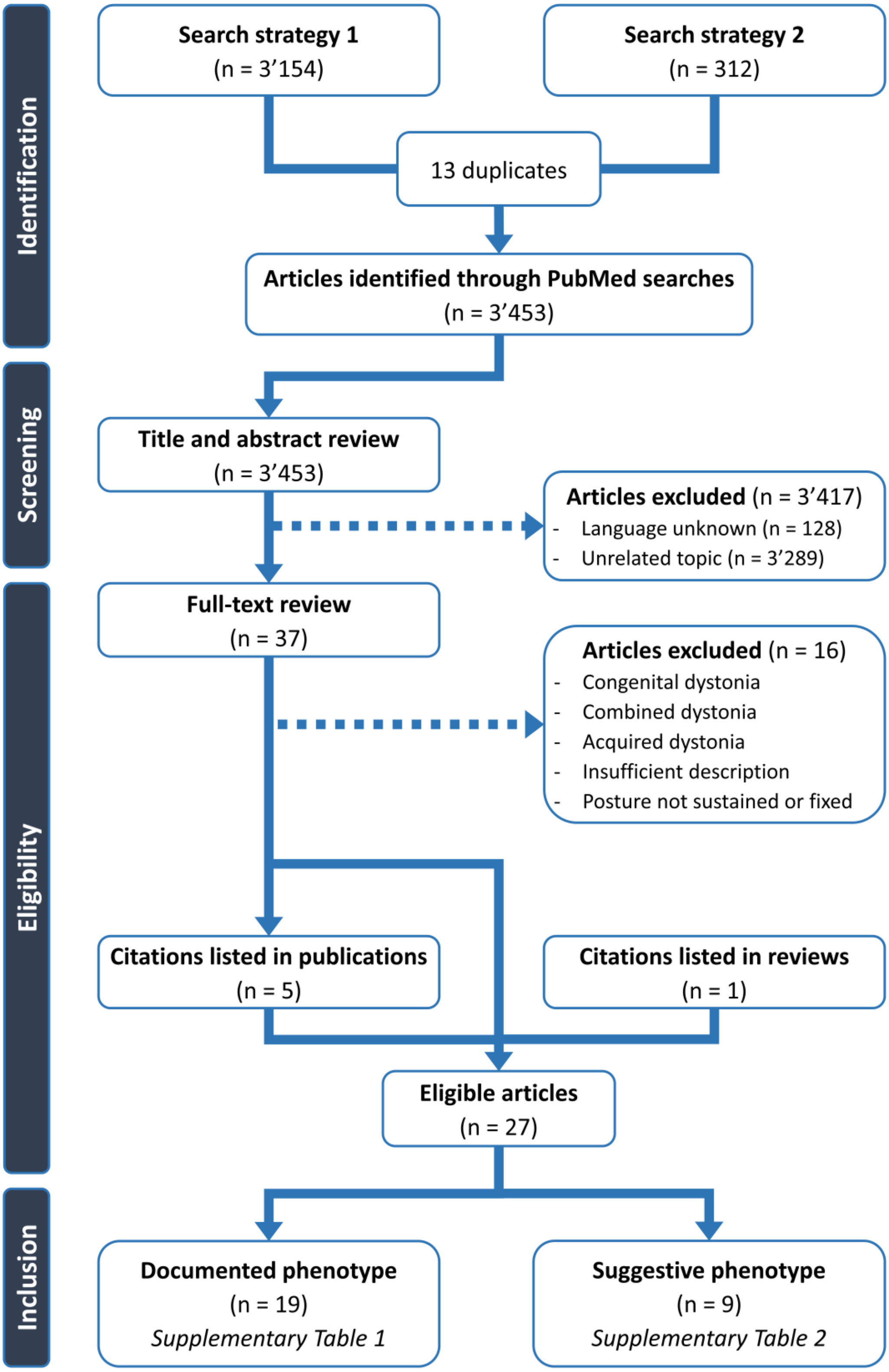

Supplementary tables

Supplementary Table 1. Characteristics of previously reported patients with sufficient clinical details to provide unequivocal recognition of sustained shoulder elevation posture.

Patients (age, sex)	Reported phenomenology	Pain	Precipitant	Onset and course	Treatments	Ref.
Case 1 (39, M)	Right torticollis, left laterocollis, anterior shift, left shoulder elevation. Alleviating manoeuvres not reported. Reduced AROM.	Neck, shoulder, and throat	Blow to the neck.	Acute onset; stable course for 4 years.	Effective: BoNT.<br>Ineffective: benzodiazepines, anticholinergics, cervical laminectomy and arthrodesis.	1
Case 3 (27, F)	Right laterocollis, right shoulder elevation. Alleviating manoeuvres not reported. Reduced AROM.	Not reported	Head and neck struck against the door of a dishwasher, no loss of consciousness.	Acute onset; course not specified.	Partially effective: BoNT.<br>Ineffective: trihexyphenidyl, clonazepam.	1
Case 4 (19, M)	Right torticollis, left laterocollis; left shoulder elevation. Alleviating manoeuvres not reported. Reduced AROM.	Neck	Minor head trauma.	Acute onset; stable course for 38 years.	Partially effective: BoNT, multi-level posterior cervical rhizotomy.<br>Ineffective: trihexyphenidyl, clonazepam.	1
Case 2 (40, M)	Sustained left laterocollis and antecollis, right shoulder elevation. Alleviating manoeuvres not reported. No cervical AROM or PROM. Muscle hypertrophy.	Neck	Street accident with mild cervical trauma.	Acute onset; course not specified; resolution in <3 years.	Not reported.	2
Case 1 (36, M)	Sustained left shoulder elevation and left laterocollis. Alleviating manoeuvres not reported. Hypertrophy of trapezius muscle.	Neck and shoulder	Traction injury of neck and shoulder.	Acute onset; stable course; resolution in 3.5 years.	Ineffective: BoNT.	3
Case 2 (27, F)	Sustained left shoulder elevation, sustained left laterocollis. Alleviating manoeuvres not reported. Hypertrophy of trapezius.	Neck and shoulder	Traction injury of the shoulder.	Acute onset; stable course; resolution in 3.75 years.	Ineffective: BoNT.	3
Case 4 (35, F)	Left shoulder elevation, sustained left laterocollis. Alleviating manoeuvres not reported. Hypertrophy of trapezius.	Neck	Lifting a heavy weight.	Acute onset; stable course; resolution in 8 years.	Partially effective: BoNT.	3
Case 5 (30, F)	Left shoulder elevation, head in the neutral position. Alleviating manoeuvres not reported. Hypertrophy of trapezius.	Neck	MVA (whiplash).	Acute onset; stable course; resolution in 3 years.	Ineffective: BoNT.	3
Case 6 (42, F)	Sustained left shoulder elevation and left	Neck and	Jaw trauma following	Acute onset; stable	Partially effective: BoNT.	3

	laterocollis. Alleviating manoeuvres not reported. Hypertrophy of trapezius.	jaw	an assault.	course; resolution in 5 years.		
Case 11 (35, F)	Right laterocollis; right shoulder elevation. Reduced PROM.	Neck	MVA (whiplash).	Gradual onset; progressive course; resolution in 8 years.	<u>Partially effective:</u> BoNT.	3
Case 13 (43, M)	Left torticollis; left laterocollis; left shoulder elevation. Efficacious alleviating manoeuvre.	Neck	Unspecified head trauma during a fall.	Gradual onset; progressive course; resolution in 13 years.	<u>Partially effective:</u> BoNT.	3
Case 14 (61, M)	Left laterocollis; left shoulder elevation. Efficacious alleviating manoeuvres.	Neck	Unspecified MVA.	Gradual onset; progressive course; resolution in 8 years.	<u>Partially effective:</u> BoNT.	3
Case 1 (36, M)	Left shoulder elevation. Hypertrophy of left trapezius.	Shoulder	Trauma to both shoulders by falling pipes.	Acute onset; stable course for 2 years.	<u>Ineffective:</u> bed rest, physical therapy, BoNT.	4
Case 2 (32, F)	Left shoulder elevation and anterior rotation; mild scoliosis and truncal rotation to the right. Muscle hypertrophy.	Shoulder	Shoulder trauma during MVA.	Acute onset; course not specified.	<u>Partially effective:</u> BoNT.	4
Case 1 (46, M)	Sustained left shoulder elevation. Efficacious alleviating manoeuvres. Hypertrophy of trapezius.	Shoulder, head and neck	Fall from a bike.	Acute onset; relapsing course; persistence for 14 months.	<u>Partially effective:</u> BoNT.	5
Case 2 (32, M)	Sustained right shoulder elevation. Hypertrophy of trapezius.	Shoulder	Ground impact with a shoulder.	Onset not reported; stable course for 8 years.	<u>Partially effective:</u> BoNT.	5
13 cases (39 ±14, 8 M, 5 F)	Sustained unilateral elevation of one shoulder in 12 patients; bilateral shoulder elevation in patient 3. Efficacious alleviating manoeuvre in one patient. Hypertrophy of trapezius. Reduced shoulder AROM.	Shoulder	Minor Injury to the shoulder girdle or neck region, 5 patients had MVA (9 patients).	Acute onset with stable course in most patients. Progressive course with generalization in one patient after a second MVA.	<u>Partially effective:</u> BoNT (six patients treated).<br><u>Ineffective:</u> oral medications for dystonia.	6
13 cases (44, ±6, 7 M, 6 F)	Sustained elevation of the left shoulder in six patients, of the right shoulder in five. Ipsilateral hypertrophy of trapezius; laterocollis and torticollis toward the elevated shoulder in nine. Alleviating manoeuvres not reported.	Cervical	Cervical disc surgery for disc herniation.	Unspecified.	Not reported.	7
Case 1 (40, M)	Right laterocollis, right shoulder elevation, right elbow flexion. Alleviating manoeuvres not reported. Fine tremor. No PROM. Hypertrophy of neck muscles.	Shoulder	MVA.	Acute onset; stable course.	<u>Effective:</u> General anaesthesia, amytal.<br><u>Ineffective:</u> BoNT.	8
Case 2 (23, M)	Right laterocollis; right shoulder elevation. Alleviating manoeuvres not reported. No PROM. Hypertrophy of neck	Shoulder	Trauma to the left shoulder.	Acute onset; progressive course in the first weeks then stable.	<u>Effective:</u> general anaesthesia, amytal.	8

	muscles.					
Case 3 (37, M)	Sustained right laterocollis; Right shoulder elevation. Alleviating manoeuvres not reported. No PROM. Hypertrophy of neck muscles.	Shoulder and neck	MVA.	Acute onset; stable course.	Effective: general anaesthesia, amytal.	8
Case 4 (29, F)	Sustained right laterocollis; right shoulder elevation; left shoulder depression. Alleviating manoeuvres not reported. No PROM. Hypertrophy of neck muscles.	Shoulder	Lifting heavy objects.	Acute onset; progressive course over two days, then stable.	Effective: general anaesthesia. Partially effective: amytal.	8
Case 5 (41, M)	Sustained left laterocollis; left shoulder elevation. Alleviating manoeuvres not reported. No PROM. Hypertrophy of neck muscles.	Shoulder and neck	MVA.	Acute onset; progressive course over one week, then stable.	Partial effective: general anaesthesia. Ineffective: BoNT.	8
Case 6 (28, M)	Sustained right laterocollis; right shoulder elevation. Alleviating manoeuvres not reported. No PROM. Hypertrophy of neck muscles.	Arm, shoulder, and neck	MVA.	Acute onset; progressive course within two weeks, then stable.	Effective: amytal.	8
Case 7 (58, F)	Sustained right laterocollis; right shoulder elevation. Alleviating manoeuvre not reported. Inconsistent gait; distractible tremor. No PROM. Hypertrophy of neck muscles.	Shoulder, neck and back	MVA.	Acute onset; progressive course over three days, then stable.	Effective: amytal.	8
Case 8 (20, F)	Sustained right laterocollis; right shoulder elevation. Alleviating manoeuvres not reported. Quasi-rhythmic head tremor; inconsistent hand tremor. No PROM. Hypertrophy of neck muscles.	Neck and shoulder	Lifting a heavy object.	Acute onset; relapsing remitting course.	Partially effective: amytal. Ineffective: BoNT, anticholinergics.	8
Case 9 (32, F)	Sustained right laterocollis; right shoulder elevation. Alleviating manoeuvres not reported. Inconsistent gait. No PROM. Hypertrophy of neck muscles.	Shoulder and back	Repetitive strain.	Acute onset, gradual progression.	Effective: amytal.	8
Case 10 (30, F)	Sustained left laterocollis; left shoulder elevation; hand posturing. Alleviating manoeuvres not reported. Variable visual field defect. No PROM. Hypertrophy of neck muscles.	Neck and shoulder	Undetailed fall.	Acute onset; progressive course over two weeks, then stable.	Effective: saline solution, amytal.	8
Case 11 (29, F)	Sustained left laterocollis; left shoulder elevation. Efficacious alleviating manoeuvre. No PROM. Hypertrophy of neck muscles.	Neck, shoulder and jaw	Undetailed fall.	Acute onset; progressive course over days, then stable.	Partially effective: BoNT.	8

Case 13 (36, M)	Sustained left laterocollis, left shoulder elevation. Alleviating manoeuvres not reported. No PROM. Hypertrophy of neck muscles.	Neck, ear and shoulder	Undetailed MVA.	Acute onset; progressive course over few days, then stable.	Not reported.	8
Case 14 (28, F)	Sustained right laterocollis, right shoulder elevation. Alleviating manoeuvres not reported. No ROM. Hypertrophy of neck muscles.	Neck, shoulder and upper back	Undetailed MVA.	Acute onset; gradual course, then stable.	<u>Partial effective:</u> amytal.	8
Case 15 (35, M)	Sustained right laterocollis, right shoulder elevation. Alleviating manoeuvre not reported. No PROM Hypertrophy of neck muscles.	Neck, shoulder, hand	Undetailed work accident.	Acute onset; progressive course over few days, then stable.	<u>Effective:</u> amytal.	8
Case 16 (18, M)	Sustained right laterocollis, right shoulder elevation, fingers clawed, hip abduction, foot posturing, paroxysmal abduction/adduction of thighs on attempt to adduct hips. Head tremor. Alleviating manoeuvres not reported. No PROM. Hypertrophy of neck muscles.	Neck, shoulder, hand, legs	MVA (whiplash).	Acute onset; progressive course after a second whiplash event.	<u>Partially effective:</u> BoNT, amytal.	8
Case 1 (32, M)	Sustained right laterocollis and right shoulder elevation; right arm pressed to the body and flexed at the elbow and wrist; finger flexion. Sustained head posture. Alleviating manoeuvres not reported. No APROM. Hypertrophy of the right sternocleidomastoid.	Neck, shoulder, arm	Cervical trauma (hit on the back of the neck).	Gradual onset; progressive course over one year; later CPRS developed.	<u>Ineffective:</u> steroid, physical therapy, BoNT, cervical epidural infusion of anaesthetics drugs, selective denervation, stellate ganglion block, right thoracic sympathectomy, and right brachial plexus decompression.	9
Case 8 (45, M)	Left torticollis, right laterocollis, retrocollis, left shoulder elevation. Reduced PROM. Alleviating manoeuvres not reported. Hypertrophy of the right sternocleidomastoid, left splenius capitis, and trapezius muscle. CRPS in the upper limb.	Neck	Quick twist of the head to the left.	Gradual onset; stable course.	<u>Partially effective:</u> BoNT, selected posterior ramisectomy (C1-C5) with myectomy of the levator scapulae, resection of the levator scapulae and splenius capitis.<br><u>Ineffective:</u> anticholinergics, neuroleptics, baclofen; general anaesthesia.	9
Case 7 (33, F)<br>Case 8 (41, F)<br>Case 9 (35, F)<br>Case 11 (39, F)<br>Case 12 (36, F)	Left shoulder elevation and sustained left laterocollis (2 patients), sustained right laterocollis and right shoulder elevation (3 patients), right laterocollis and right	Not specified	MVA (whiplash).	Acute onset; symptom duration 2-15 years.	<u>Effective:</u> BoNT in some patients.	10

Case 14 (18, F)	shoulder elevation and subsequent truncal dystonia (1 patient). Efficacious alleviating manoeuvres in 4 patients. Hypertrophy of trapezius (all patients).					
Case 4 of prospective cohort (31, M)	Sustained left shoulder elevation.	Neck	Neck surgery.	Onset not specified; stable course; no remission over 8 years.	Not specified.	11
Case 1 (34, F)	Sustained shoulder elevation; left laterocollis; left foot supinated and adducted; left knee hyperextended. Alleviating manoeuvre not reported. Type 1 CRPS in the left foot.	Left foot	MVA (fracture of the third metatarsal bone of left foot, followed by casting.	Onset not specified; progressive course involving the shoulder.	Partially effective: opiates, spinal anaesthesia.<br>Ineffective: BoNT, anticholinergics, right GPe DBS, ventralis lateralis posterior DBS, GPi DBS.	12
Case 1 (24, M)	Left shoulder elevation. Full AROM, PROM. Rightward scoliotic deviation of the spine. Hypertrophy of the left trapezius.	Shoulder	Fell down 3 meters while wearing a Kevlar body armour and carrying an heavy backpack.	Subacute onset; stable course.	Partially effective: BoNT.	13
Case 1 (45, M)	Left shoulder elevation; right torticollis; retrocollis. Efficacious alleviating manoeuvres. Chronic spasms of paraspinal muscles, left trapezius and sternocleidomastoid.	Neck	C3-C4 myelopathy followed by cervical anterior corpectomy and halo-vest application for 6 weeks.	Subacute onset; progressive course over the first two months.	Effective: BoNT.<br>Ineffective: diazepam, tizanidine hydrochloride, trihexyphenidyl.	14
Case 1 (31, F)	Right shoulder elevation; sustained elevation and anterior rotation of the left shoulder; abduction of the upper limb; clenched fist; sustained kyphoscoliosis. Alleviating manoeuvres not reported.	Shoulder	Not reported.	Gradual onset; progressive course.	Partially effective: motor cortex stimulation, deep sedation.<br>Ineffective: benzodiazepine, baclofen, trihexyphenidyl, BoNT, left scalene myectomy, bilateral GPi DBS.	15
Case 1 (22, M)	Right shoulder elevation. No alleviating manoeuvres. AROM reduced, PROM unimpaired.	Shoulder, arm and neck	Fall on the right shoulder against a post.	Gradual onset; resolution after 4 months.	Partially effective: physical therapy.	16
Case 3 (NA, M)	Left shoulder elevation. Hypertrophy of trapezius. Alleviating manoeuvres not reported.	Shoulder	Undetailed peripheral trauma.	Acute onset; resolution in <24 hours.	Not reported.	17
Case 4 (NA, M)	Right shoulder elevation. Hypertrophy and spasm of the right trapezius.	Not reported	Undetailed MVA.	Gradual onset; persistence for >9 months.	Not reported.	17
Case 1 (17, M)	Left shoulder elevation; lateral trunk tilt; right laterocollis and immobility of proximal right arm. Alleviating manoeuvres not reported. CRPS in the right arm.	Shoulder	Violent blow to the right shoulder while playing football.	Acute onset; relapsing course after steroid withdrawal and onset of	Effective: intravenous methylprednisolone.<br>Partially effective: oxycodone.<br>Ineffective: non-steroidal anti-	18

				CRPS in right arm; remission after a second steroid trial without relapse over six months.	inflammatories, metaxalone, carbidopa/levodopa, trihexyphenidyl baclofen, physical therapy.	
Case 1 (40, M)	Sustained left shoulder elevation; dystonic movements of the left shoulder. Reduced shoulder AROM and PROM. Hypertrophy of trapezius.	Upper limb and shoulder	Fall from motorbike over the left arm.	Gradual onset; course not reported; complete remission.	<u>Effective:</u> BoNT. <u>Partially effective:</u> analgesics, baclofen, anticholinergics, antidepressants, benzodiazepines.	19

Abbreviations: AROM, active range of motion; BoNT, Botulinum neurotoxin; DBS, Deep brain stimulation; CRPS, Complex regional pain syndrome; GPe, Globus pallidus pars externa; GPi, Globus pallidus pars interna; MVA, motor vehicle accident; PROM, passive range of motion; NA, Not available.

Supplementary Table 2. Characteristics of previously reported patients with insufficient clinical details to provide unequivocal recognition of sustained shoulder elevation posture.

Patients	Main phenomenology	Pain	Precipitant	Onset and course	Treatments	Ref.
Page 164-5 (young, M)	Jerking of head and shoulders, grimaces of the lips and face.	Not reported	Sergeant exposed to shell shock during World War 1.	Following a Zeppelin raid, activated by noise; lasted for some months.	<u>Not reported.</u>	20
Case 1 (32, F)	Sustained abduction and external rotation of the right shoulder; no active movements; winged scapula; spasm of the right deltoid. No PROM.	Shoulder	Shoulder strain while cutting grass.	Acute onset; relapsing course.	<u>Partially effective:</u> physical therapy, benzodiazepines, local heat.	21
Case 6 (42, M)	Severe retrocollis; intermittent torticollis; mild blepharospasm; mild movements of the trunk and shoulders.	Neck	Whiplash injury.	Acute onset; course not reported.	<u>Effective:</u> anticholinergics.	22
Case 4 (23, F)	Neck spasms, immobile straight neck, no AROM, PROM. Alleviating manoeuvres not reported. Hypertrophy of the left trapezius.	Neck and shoulder	MVA.	Acute onset; resolution over <3 years.	<u>Ineffective:</u> physical therapy, general anaesthetics.	2
Case 10 (28, F)*	Right shoulder dystonia at rest	Not clearly reported	MVA.	Acute onset; progression to face and right arm over one year.	<u>Partially effective:</u> placebo injection. <u>Ineffective:</u> amytal.	23
Case 1 (39, M)	Increased muscle bulk in the left trapezius with impaired shoulder control and arm abduction. Normal PROM.	Not reported	Removal of a lipoma posteriorly in the neck.	Gradual onset; no progression over six months.	<u>Partially effective:</u> BoNT.	24
Case 1 (NA)	Laterocollis, retrocollis, and right shoulder elevation.	Not reported	Not reported.	Not reported.	Not reported.	25
Case 4 (17, F)	CRPS associated with sustained posture	Allodynia involving	Dog bite to the right leg.	Acute onset; progression over few months with	<u>Partially effective:</u> cervical sym-	26

	of the right arm: shoulder internally rotated, elbow flexed and pronated, with fisting and flexion at wrist, right hemifacial spasm. Alleviating manoeuvres not reported.	right hand and forearm		right foot involvement.	pathetic block, local sympathetic blocks in the right upper and lower limbs, BoNT, casting of right upper limb. Temporarily effective: unspecified psychiatric treatment.	
Case 2 (22, M)	Left shoulder elevation; torticollis to the right; mild retrocollis.	Not reported	No trauma.	Not reported.	Partially effective: BoNT.	27
Case 3 (6, F)	Severe torticollis with right shoulder elevation.	Not reported	Unspecified cervical trauma; C2-C3 subluxation.	Not reported.	Not reported.	27

* This description may correspond to case 14 published by the same team some years later (included in supplementary table 1) [8]. Abbreviations: AROM, Active range of motion; CRPS, Complex regional pain syndrome; MVA, motor vehicle accident; NA, Not available; PROM, Passive range of motion.

References

1. Truong DD, Dubinsky R, Hermanowicz N, Olson WL, Silverman B, Koller WC. Posttraumatic torticollis. *Arch Neurol*. Feb 1991;48(2):221-3. doi:10.1001/archneur.1991.00530140117025

2. Goldman S, Ahlskog JE. Posttraumatic cervical dystonia. *Mayo Clin Proc*. May 1993;68(5):443-8. doi:10.1016/s0025-6196(12)60192-x

3. Tarsy D. Comparison of acute- and delayed-onset posttraumatic cervical dystonia. *Mov Disord*. May 1998;13(3):481-5. doi:10.1002/mds.870130318

4. Thyagarajan D, Kompoliti K, Ford B. Post-traumatic shoulder 'dystonia': persistent abnormal postures of the shoulder after minor trauma. *Neurology*. Oct 1998;51(4):1205-7. doi:10.1212/wnl.51.4.1205

5. Hollinger P, Burgunder J. Posttraumatic focal dystonia of the shoulder. *Eur Neurol*. 2000;44(3):153-5. doi:10.1159/000008225

6. Wright RA, Ahlskog JE. Focal shoulder-elevation dystonia. *Mov Disord*. Jul 2000;15(4):709-13. doi:10.1002/1531-8257(200007)15:4<709::aid-mds1017>3.0.co;2-4

7. Becker G, Berg D, Kruse N*, et al*. Evidence for shoulder girdle dystonia in selected patients with cervical disc prolapse. *Mov Disord*. Jul 2002;17(4):710-6. doi:10.1002/mds.10132

8. Sa DS, Mailis-Gagnon A, Nicholson K, Lang AE. Posttraumatic painful torticollis. *Mov Disord*. Dec 2003;18(12):1482-91. doi:10.1002/mds.10594

9. Frei KP, Pathak M, Jenkins S, Truong DD. Natural history of posttraumatic cervical dystonia. *Mov Disord*. Dec 2004;19(12):1492-8. doi:10.1002/mds.20239

10. O'Riordan S, Hutchinson M. Cervical dystonia following peripheral trauma--a case-control study. *J Neurol*. Feb 2004;251(2):150-5. doi:10.1007/s00415-004-0291-9

11. Schrag A, Trimble M, Quinn N, Bhatia K. The syndrome of fixed dystonia: an evaluation of 103 patients. *Brain*. Oct 2004;127(Pt 10):2360-72. doi:10.1093/brain/awh262

12. Capelle HH, Grips E, Weigel R*, et al*. Posttraumatic peripherally-induced dystonia and multifocal deep brain stimulation: case report. *Neurosurgery*. Sep 2006;59(3):E702; discussion E702. doi:10.1227/01.NEU.0000229000.28373.95

13. Carroll CG, Hawley JS, Ney JP. Post-traumatic shoulder dystonia in an active duty soldier. *Mil Med*. Jun 2006;171(6):494-6. doi:10.7205/milmed.171.6.494

14. Takemoto M, Ikenaga M, Tanaka C, Sonobe M, Shikata J. Cervical dystonia induced by cervical spine surgery: a case report. *Spine (Phila Pa 1976)*. Jan 1 2006;31(1):E31-4. doi:10.1097/01.brs.0000193928.16048.cb

15. Romito LM, Franzini A, Perani D*, et al*. Fixed dystonia unresponsive to pallidal stimulation improved by motor cortex stimulation. *Neurology*. Mar 13 2007;68(11):875-6. doi:10.1212/01.wnl.0000256816.83036.c9

16. Drouet A, Have L, Jacquin O, Guilloton L, Felten D. Dystonie focale fixée de l'épaule à distance d'un traumatisme scapulaire minime : une entité pour quels mécanismes? Post-traumatic focal fixed dystonia of the shoulder: a distinctive syndrome with speculative mechanisms? *Rev Neurol (Paris)*. Nov 2009;165(11):975-9. doi:10.1016/j.neurol.2008.10.025

17. Jankovic J. Peripherally induced movement disorders. *Neurol Clin*. Aug 2009;27(3):821-32, vii. doi:10.1016/j.ncl.2009.04.005

18. Hassan A, Pittock SJ, Ahlskog JE. Steroid-responsive post-traumatic dystonia: a video case report. *Parkinsonism Relat Disord*. Feb 2011;17(2):130-2. doi:10.1016/j.parkreldis.2010.10.009

19. Vasileiadis GI, Sakellariou VI, Papagelopoulos PJ, Zoubos AB. Posttraumatic focal dystonia of the shoulder. *Orthopedics*. Jun 2012;35(6):e977-80. doi:10.3928/01477447-20120525-47

20. Mott FW. *War neuroses and shell shock*. H. Frowde; Hodder & Stoughton; 1919.

21. Innes AR, MacNicol MF. Nonprogressive abduction contracture of the shoulder joint. A case report. *Clin Orthop Relat Res*. Jun 1986;(207):205-8.

22. Schott GD. Induction of involuntary movements by peripheral trauma: an analogy with causalgia. *Lancet*. Sep 27 1986;2(8509):712-6. doi:10.1016/s0140-6736(86)90231-x

23. Lang AE. Psychogenic dystonia: a review of 18 cases. *Can J Neurol Sci*. May 1995;22(2):136-43. doi:10.1017/s031716710004021x

24. Cossu G, Melis M, Melis G, Ferrigno P, Molari A. Persistent abnormal shoulder elevation after accessory nerve injury and differential diagnosis with post-traumatic focal shoulder-elevation dystonia: report of a case and literature review. *Mov Disord*. Sep 2004;19(9):1109-11. doi:10.1002/mds.20142

25. Garcia Ruiz PJ, Martinez Castrillo JC. Cervical dystonia and Constantin Brancusi. *Mov Disord*. Sep 15 2009;24(12):1849-50. doi:10.1002/mds.22684

26. Majumdar A, Lopez-Casas J, Poo P, *et al*. Syndrome of fixed dystonia in adolescents--short term outcome in 4 cases. *Eur J Paediatr Neurol*. Sep 2009;13(5):466-72. Not in File. doi:10.1016/j.ejpn.2008.09.005

27. Raju S, Ravi A, Prashanth LK. Cervical Dystonia Mimics: A Case Series and Review of the Literature. *Tremor Other Hyperkinet Mov (N Y)*. 2019;9doi:10.7916/tohm.v0.707
